# Delivering and implementing child and adolescent mental health training for mental health and allied professionals: a systematic review and qualitative meta-aggregation

**DOI:** 10.1186/s12909-021-02530-0

**Published:** 2021-02-15

**Authors:** Emily Banwell, Neil Humphrey, Pamela Qualter

**Affiliations:** Institute of Education, University of Manchester, Ellen Wilkinson Building, Manchester, M13 9PL UK

**Keywords:** Training, Mental health, Children, Adolescents, Young people, Professional development, Implementation science, Qualitative, Systematic review, Meta-aggregation

## Abstract

**Background:**

The increasing prevalence of mental health difficulties among children and young people (CYP) suggests that early intervention is vital. A comprehensive system of care and support requires the involvement of mental health professionals, including psychologists and psychiatrists, and allied professionals, including teachers, police, and youth workers. A critical starting point is the provision of effective training, in order that these professionals can better support the mental health needs of the CYP that they encounter.

**Objectives:**

Given the primacy of training in the CYP mental health support system, understanding the factors that maximise potential gains and facilitate uptake is pertinent. The current review therefore located and explored qualitative research evidence, to identify the barriers and facilitators underpinning successful delivery and implementation of training focussed on the mental health of CYP, for both mental health and allied professionals.

**Methods:**

A systematic review and qualitative meta-aggregation were conducted. Systematic searches were carried out using ASSIA, EMBASE, MEDLINE, NICE Evidence, PsycINFO, and Scopus databases, for papers published between 2000 and 2020. Twelve thousand four hundred forty-eight records were identified, of which 39 were eligible for review. The records were appraised for quality using the Joanna Briggs Institute Critical Appraisal Checklist for Qualitative Research, and synthesised using the qualitative meta-aggregation method.

**Results:**

One hundred eighty-two raw findings were extracted from the 39 papers, which were condensed into 47 sub-categories, 19 categories, and finally 5 synthesis statements. These synthesis statements reflected the barriers and facilitators influencing the training delivery process (“support”; “content, design, and planning”), and the implementation of training into the workplace (“context”; “perceived value”; “organisational factors”).

**Conclusions:**

The synthesis statements and underlying categories provide practical recommendations for those designing, delivering, or implementing CYP mental health training. Recommendations ranged from facilitating peer support during training, to the idea that training will be better implemented when perceived need is high. The review provides a robust evidence-based foundation to “common-sense” principles, drawing them into a coherent and organised framework using a synthesis method grounded in pragmatism.

**Protocol registration number:**

PROSPERO reference ID: CRD42020162876.

**Supplementary Information:**

The online version contains supplementary material available at 10.1186/s12909-021-02530-0.

## Introduction

### Rationale

Mental health difficulties are common among children and young people (CYP). In 2020, one in six 5–16 year-olds (16%) in England had a probable diagnosable disorder [1]. This pattern is similar worldwide, with 10–20% of children and adolescents experiencing mental ill health [2]. Looking at incidence across the lifespan, half of all psychiatric conditions start before the age of 14 [2, 3]. Mental health difficulties are associated with multiple salient individual and societal outcomes. Longitudinal studies have found associations between poor mental health in childhood and adolescence, and lower quality of life and loneliness [4], higher criminal behaviour [5], and poorer health, both mental and physical, in adulthood [4, 6]. In terms of financial burden, mental ill health is estimated to cost over £100 billion in England per year [7] when the detrimental impact on economic productivity is considered alongside higher service utilisation [8]. These statistics clearly suggest that early intervention is vital if poor mental health among CYP is to be ameliorated.

Mental health professionals, such as psychologists, psychiatrists, and mental health nurses immediately come to mind when we consider those who provide relevant care and support for CYP. High quality training, whether basic or specialist, should be offered actively and regularly to these professionals, to improve and update their skills and knowledge [9]. However, CYP also encounter a wide variety of non-mental health trained professionals in their everyday lives, including teachers, police, and general healthcare providers. These allied professionals also need to be well placed to support the needs of the CYP they encounter, irrespective of whether or not they have been referred to specialist mental health services.

A climate of austerity and budget cuts to Child and Adolescent Mental Health Services (CAMHS) in the UK means that these alternative, non-specialised services are increasingly being relied upon to provide mental health support. In 2014/2015, only 25% of CYP with a psychiatric disorder had made contact with mental health services [10], compared to 38% in 2005/2006 [11]. A funding cut of 5.4% to CAMHS within this period is perhaps responsible for this severely reduced rate of service contact [12]. Additionally, an increase in average waiting times for access to CAMHS [13], along with a substantial likelihood of referral rejection [14], means that many CYP cannot benefit from specialist support, particularly when the cross-sector communication needed to signpost to alternative sources of support is notoriously poor [15]. Indeed, a recent systematic review found that over 25% of CYP with diagnoses or elevated symptoms were not utilising *any* form of mental health support, specialist or otherwise [16], suggesting that even alternative support is inaccessible to many.

Bearing these issues in mind, teachers were found to be the most common allied service contact that CYP and/or their parents utilised regarding emotional, behavioural, or concentration difficulties [17]. Teachers perceive themselves as being the “front line” for help-seeking for several reasons, including the close bonds forged throughout the school year, and the mental health stigma held by some parents [18]. However, because mental health support is not the primary role of school staff, they do not have the time and resources needed to effectively provide it, nor do they feel adequately trained to do so [18]. Even primary medical professionals such as general practitioners feel under-equipped to recognise issues and provide appropriate support for CYP, with the criteria for CAMHS referral poorly understood [19]. Reduced government funding for mental healthcare means that patients of all ages are more likely to turn to Accident and Emergency (A&E) departments at times of crisis [20]. For CYP specifically, between 2010 and 2015, the number of psychiatric A&E attendances doubled [21]. A&E staff perceive their own knowledge and effectiveness for dealing with CYP psychiatric admissions as low [22], with the A&E environment decidedly unsuitable to care for such patients [20].

Clearly, CYP are clearly “falling between the gaps” in terms of accessing the support they need. Recent efforts have consequently been made to ensure that CYP mental health is “everybody’s business”. Initiatives such as i-THRIVE [23], introduced in 70 areas in England, emphasise the value of providing support through a diverse range of access points, not only health services (e.g. the UK’s National Health Service). i-THRIVE represents a shift from a mind-set where mental health is solely the purview of health professionals, to one where schools, social care, and even the arts sector, can be informed advisors - providing support, and signposting effectively and confidently [23]. For this vision to become a reality, training a diverse range of allied professionals should be a priority. They should be equipped with the skills required to provide appropriate support, be this individualised or community-based care. The latter, for example school-based mental health promotion [24] can benefit even healthy populations of CYP, helping them to deal with the inevitable “ups and downs” of life [23].

What should training look like? In broad terms, mental health training should improve the mental health literacy of its trainees. Mental health literacy refers to the understanding of mental health problems, how to improve mental health, and confidence in knowing when, where, and how, to provide or signpost to assistance [25]. Additionally, an increase in literacy and awareness should result in a reduction of negative stigma [26]. In terms of content, training programmes often vary in specificity, depending on the type of professionals being trained. Basic level programmes such as Youth Mental Health First Aider training [27], or basic psychotherapeutic skills [28], may be suitable for allied professionals. However, mental health professionals should be offered more focussed training that reflects their level of background knowledge [27]. Training is also one of the most widely used implementation strategies when disseminating new evidence-based practices in CYP mental health [29].

### Objectives and research questions

Given the primacy of training in the CYP mental health support system, it is vital to understand the factors that maximise its potential gains and facilitate uptake, for example, a training programme being of appropriate complexity for those completing it [28]. To date, a number of qualitative studies have explored these barriers and facilitators, by speaking to those receiving and delivering training. However, a systematic review that aggregates research across the field, where both mental health *and* allied professionals are participants, has not yet been conducted.

Considering this, the current review located and explored relevant qualitative research evidence, to identify the barriers and facilitators underpinning successful delivery and implementation of training that focusses on the mental health of CYP. These barriers and facilitators were established by collating the experiences and views of mental health trained professionals, along with any allied professionals who might, in their daily roles, encounter CYP who require mental health support. The review built upon the findings of a similar qualitative review by Scantlebury et al. (2018) [30]. Their qualitative synthesis identified several delivery and organisational factors, reported by allied professionals, as predictive of whether or not training was well implemented. By simultaneously narrowing the reach of the systematic search to only include studies of training pertaining to CYP, and broadening it to include the views of mental health professionals, the current review sought to provide further insights into how training delivery, and its subsequent implementation in practice, could be improved for all professionals. Given that such a wide range of professionals are currently so closely involved with supporting CYP, even those with diagnosed psychiatric disorders, it may be the case that their experiences do not vary as much as one would immediately imagine. As such, the current review was able to explore whether, and if so, how, the reported barriers and facilitators differed by professional group.

The review sought to answer the following questions:

1. What are the barriers and facilitators that a) mental health professionals, and b) allied professionals, perceive as influencing the training delivery process?
2. What are the barriers and facilitators that a) mental health professionals, and b) allied professionals, perceive as influencing the implementation of training in the workplace?
3. Based on the above, what evidence-based recommendations can be made in order to improve training delivery and implementation?

## Methods

A systematic review and qualitative meta-aggregation were conducted, following the Preferred Reporting Items for Systematic Reviews and Meta-Analyses (PRISMA) guidelines.

### Protocol

The methodology and inclusion and exclusion criteria for the review were published in a PROSPERO protocol in January 2020 (reference: CRD42020162876). Progress updates were documented periodically.

### Eligibility criteria

The inclusion and exclusion criteria that were used to decide if a paper was eligible for the review can be found in Table 1*.*

**Table 1:** Inclusion and exclusion criteria

Inclusion:	Exclusion:
*Topic:* • Training or staff development programmes focussed on responding to, and/or improving knowledge of, the mental health of CYP	• Training to improve professionals’ own mental health • Studies focussed on all-age mental health where the data pertaining to CYP is not separable • Studies where training focusses heavily on another topic (e.g. child protection, physical health, behavioural management), and any data on mental health is not separable
*Sample:* • Mental health or allied professionals as defined above, who received and/or provided the training programme	• Students of university or college courses that did not involve contact with CYP
*Design, methods, and analysis:* • All qualitative study designs • Mixed methods study designs, providing that the qualitative element was entirely separable from any quantitative analysis	• Studies with no separable qualitative findings • Studies where qualitative data collection methods were used, but qualitative analytic methods were not
*Study aims:* • Studies that explored the perceived barriers and facilitators of training delivery and/or implementation	
*Other:* • Studies with at least one extractable qualitative finding, to allow inclusion into the meta-synthesis	• Studies unavailable in the English language • Studies published prior to the year 2000 • Research protocols or conference abstracts for which a full study write-up could not be located

In this review, the distinction between the two professional types was made as follows. “Mental health professionals” provide targeted mental health interventions, ranging from early assessment and support, through to disorder-specific services and inpatient units. They include, for example, mental health nurses, psychiatrists/psychologists, and therapists.

The term “allied professionals” refers to those who, as part of their job role, are likely to encounter CYP requiring mental health support, however their role is not specialised towards providing this. They include, for example, teachers, police, or youth workers, plus medical professionals who do not specialise in mental health, such as GPs or paediatric nurses. Students were included under the two definitions, provided that their course was sufficiently vocational (e.g. trainee clinical psychologists, trainee social workers).

It was anticipated that studies might not explicitly specify the ages of the CYP that the training focussed on, instead using broader descriptors such as “children”, “adolescents”, “young people”, etc. Such studies were considered for inclusion. However, where an age bracket *was* mentioned, the review included studies focussed on training pertaining to those up to, and including, the age of 24. Despite debate around the age at which an individual is considered an adult, this age corresponds with the WHO’s definition of a “young person” [31], as well as with a recently proposed developmentally appropriate definition of “adolescence” [32].

### Search strategy

The search strategy was initially developed by the first author, based upon methodological guidance and prior SLRs. The second and third authors were consulted periodically, in order to develop the strategy in an iterative manner. The strategy included terms relating to a) staff roles (e.g. “practitioner”; “teacher”), b) mental health (e.g. “depression”; “crisis”), c) CYP (for example “teen”; “young offender”), d) training (e.g. “professional development”; “learning package”), e) study aims (e.g. “evaluation”; “experiences”), and f) data generation (e.g. “qualitative”; “interview”).

The search terms were adapted as per the requirements of each bibliographic database. See supplementary materials for a complete example of a search strategy.

### Data sources

The following databases were searched: ASSIA, EMBASE, MEDLINE, NICE Evidence, PsycINFO, and Scopus. In addition to this, the reference lists of eligible studies and relevant review articles were hand-searched for any further studies. Studies in a variety of formats were considered for inclusion, including peer-reviewed journal papers, doctoral theses, unpublished research, and conference papers, providing that extractable information was available. This approach mitigates the issue of publication bias, where the findings of peer-reviewed studies might differ substantially from those of unpublished research [33]. The years 2000 to present (January 2020) were used as date parameters in each search, minimising the chance of research findings being outdated. Whilst studies prior to 2000 may indeed be relevant, this 20-year period was deemed sufficient in order to capture research findings that were contemporary, and reflective of recent policy in relation to the mental health of CYP.

### Study selection

Results from initial searches were uploaded to Endnote, and duplicate hits were removed. The paper titles and abstracts were screened manually by the first author, and those that appeared to adhere to the inclusion criteria were retained. The third author independently replicated 10% of the screening, to check the clarity of the eligibility criteria. Discrepancies and uncertainties were discussed, and the criteria clarified as a result. Full text versions of the retained papers were then assessed by the first author against the full inclusion and exclusion criteria. 10% of these were independently reviewed by the third author to ensure consistency in approach. An agreement rate of 96.7% suggests strong replicability of the search strategy.

### Quality appraisal

The included papers were reviewed for quality using the Joanna Briggs Institute (JBI) Critical Appraisal Checklist for Qualitative Research [34]. Designed by the JBI, the tool was deemed a suitable fit for the chosen qualitative synthesis method (see “data synthesis” section). The tool comprises ten questions, ensuring that methodology, analysis, and interpretations do not contradict one another, and addressing researcher influence, ethical approval, participant representation, and logical flow from analyses to conclusions. Each item is given a score of one if it is evidenced in the paper, and zero otherwise, producing a total score from 0 to 10. All papers were independently checked by the first and third authors. Scores within three points’ difference of one another were said to agree. If the authors’ rating of a criterion differed by more than three points, they discussed their reasoning, and reached a consensus score together.

### Data extraction

A data extraction spreadsheet was designed based upon the papers’ key features, including research and analysis methods, and the purpose of the described training programmes. Details of the participants’ job roles, trainer or trainee status, and information pertaining to the populations of CYP that the training dealt with (e.g. healthy school populations, those with a specific mental health issue) were additionally extracted, in order to establish the representation of these characteristics across the papers.

### Data synthesis

The review took a deductive approach, with concepts of interest (barriers and facilitators) defined a-priori*,* and accounted for within the search terms*.* This left extraction and summation of the included studies as the key aim of the review and synthesis [35]. Consequently, qualitative meta-aggregation was deemed the most appropriate method of doing this. Outlined in the Joanna Briggs Institute Qualitative Assessment and Review Manual (JBI QARI), the meta-aggregative method is a qualitative evidence synthesis strategy grounded in a pragmatic philosophical stance [36]. It involves extracting the concluding findings from every included paper, and categorising them based on shared meaning. It then groups these categories further, summarising them to produce synthesised findings: practical, directive action points that can be used to guide policy and make recommendations [34]. Qualitative meta-aggregation seeks to avoid reinterpreting the conclusions drawn in the literature, instead aiming to present a synthesised version of the authors’ original intended meanings [34].

To identify a “finding” within a paper, a reviewer commonly draws from the list of themes presented in a qualitative study. However, in the current review, this strategy was problematic because presented themes were often very short and lacking detail. To gain a richer set of findings, a line-by-line examination of the papers’ results sections was conducted, extracting the authors’ concluding observations and remarks verbatim. The discussion and conclusion sections were also closely examined to capture any ideas not mentioned in the results sections. Along with each finding, a supporting extract was identified, in the form of a verbatim participant quotation. A level of credibility was then allocated to each finding, based upon the congruence between the author’s conclusion, and the participant’s voice [34].

**Fig. 1:**
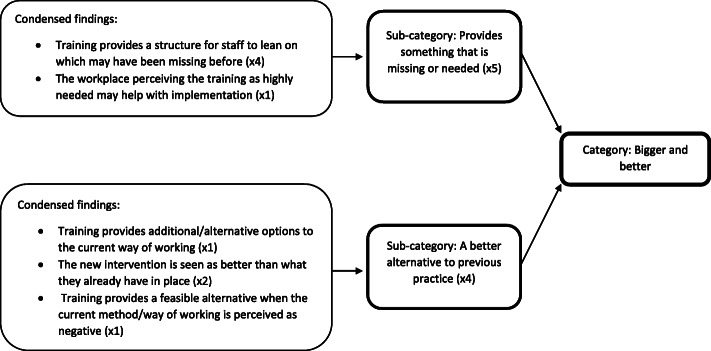
An example of the progression from condensed finding, to sub-category, to category. The numbers in brackets represent how many “raw findings” are represented by each, providing an indication of how well each category represents the data

The method of extracting concluding remarks meant that findings were numerous and often lengthy. Consequently, two additional categorisation steps were taken to produce the final synthesis statements: the production of condensed findings and sub-categories. First, a simple summative statement was produced for each finding, condensing the essence of each, and allowing more effective categorisation. Longer findings, where multiple topics were addressed, were assigned more than one condensed finding, and conversely, findings that were extremely similar were assigned to the same condensed finding. A note was made of how many raw findings were represented by each. Then, condensed findings were classified into broad categories based upon the overarching topic and meaning of the findings, before further splitting them into sub-categories. This was deemed a more appropriate method of representing the richness of the data, and was used in a qualitative meta-aggregation by Johnson & Woodgate (2017) [37]. An example of this process can be found in Fig. 1*.* Finally, the categories were grouped into synthesis statements, providing a useful broad heading for the recommendations within. Throughout the aggregation process, corresponding extracts from the papers were used to guide categorisation. The extraction of findings and data synthesis processes were carried out by the first author, however the final synthesis statements, categories, and sub-categories were discussed with the second and third authors, to gain consensus on their validity.

## Results

### Study selection and characteristics

**Fig. 2:**
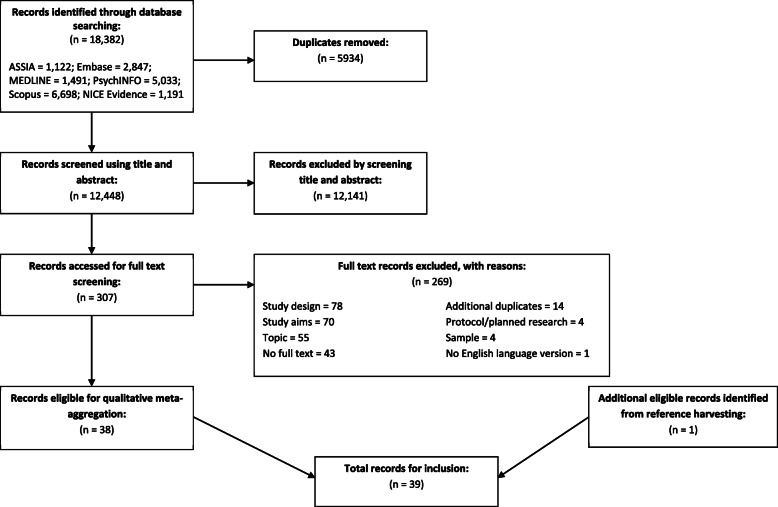
PRISMA flow diagram illustrating the process of study identification

The review process yielded 38 eligible studies from the 12,448 that were gathered from initial searches. One additional study was identified through manual checking of the reference lists of eligible studies, bringing the total number of included studies to 39. Figure 2 is the PRISMA flowchart showing the number of studies retained and excluded at each stage of the review process.

The characteristics of the 39 studies can be found in Table 2*.* Fifteen of the studies were conducted in the USA (38%), seven in the UK (18%), and six in Australia (15%). The remaining 11 studies were conducted by nine other countries, including Canada, India, Ethiopia, and Haiti. Twenty studies involved training programmes undertaken by allied professionals, with 14 involving mental health trained professionals, and five using a mixed sample. Only two studies involved trainers as participants, with 37 involving trainees.

**Table 2:** Key characteristics of included studies

Reference	Country	Type of professional	Sample (*mixed methods studies. N reflects the number of participants involved in the qualitative element)	Training programme aim(s)	CYP population training focusses on	Study aim(s)	Qualitative data collection method	Qualitative analysis method	JBI Critical Appraisal Checklist score out of 10 and (classification)
Adelman (2014) [38]	USA	Allied	Pre-kindergarten special educators (new to the field - either newly applying for jobs or in first or second year in the field) (*n* = 7)	To improve teachers’ observation skills in social responsiveness of preschool children with ASD.	Preschool children aged 4–5 years, with ASD.	To examine the potential learning outcomes for teachers using the tool to learn.	Interviews	Grounded theory coding and analysis	7 (moderate)
Askell-Williams & Murray-Harvey (2016) [39]	Australia	Allied	Early childhood educators (*n* = 1148*)	Initiative to promote mental health in young children.	General population of under 5 s.	To analyse perspectives of early childhood education and care educators’ about their professional learning.	Open ended questionnaire	Thematic analysis	7 (moderate)
Bassilios et al. (2017) [40]	Australia	Mixed	Mental health professionals (psychologists, social workers, mental health nurses) (*n* = 8); Medicare administrators (*n* = 20)	To up-skill mental health professionals to meet required skills/competencies to deliver CMHS (a new service in Australia beginning 2010).	CYP aged 11 and under who have, or are at risk of developing, psychological disorders.	To explore the utilisation of implementation processes (of which the outlined online training is one) for those CMHS that will help deliver its objectives.	Structured interviews	Theoretical semantic analysis	8 (high)
Bazyk et al. (2015) [41]	USA	Mental health	Paediatric occupational therapists and occupational therapy assistants (*n* = 117*)	A capacity building process designed to promote knowledge translation of a public health approach to mental health.	CYP having occupational therapy.	Explore the meanings and outcomes of the training.	Written reflections	Phenomenological analysis	8 (high)
Blackburn et al. (2016) [42]	Australia	Mental health	Clinical staff at a youth acute mental health inpatient unit (*n* = 18*)	To teach sensory modulation techniques to de-escalate violence and aggression in CYP mental health inpatients.	CYP aged 15–24 years, in inpatient mental health units.	To evaluate the effectiveness of the intervention in transferring knowledge to staff, and translating this knowledge into practice.	Focus groups	Thematic analysis	7 (moderate)
Bond & Dogaru (2019) [43]	UK	Mixed	Police officers, teachers, “CYP professionals”, trainee teachers, social work students, health professionals, youth workers, probation officers, family support workers, hate-crime officers, therapists, and charity Chief Executive Officer (CEOs) (n = 114*)	To develop professionals’ competence and confidence when responding to the needs of children and their families after online sexual abuse.	CYP who have been sexually abused online.	Evaluate the outcomes of the training.	Open-ended questionnaire	Thematic analysis	5 (moderate)
Bryson & Ostmeyer (2014) [44]	USA	Mental health	Social skills group leaders for children with ASD (*n* = 15*)	Group management of CYP with ASD attending social skills group.	CYP between 4 and 12 years old, who have ASD.	To look at the changes in behavioural management skills of the group leaders.	Electronic survey with free text options	Thematic analysis	7 (moderate)
Christie et al. (2013) [45]	New Zealand	Mental health	CAMHS workers (*n* = 37*)	Training to use an adolescent alcohol and drug screening and intervention.	Adolescents involved with CAMHS for whom alcohol or drugs might be an issue.	To evaluate the utility and acceptability of the training package, and its impact on relevant attitudes, skills and knowledge.	Focus groups	General inductive approach - thematic analysis	5 (moderate)
Coiro et al. (2016) [46]	USA	Mental health	Graduate student clinicians in clinical psychology, or speech-language pathology (n = 11)	To expose graduate students to inter-professional collaborative practice centred on enhancing children’s social communication.	USA school-aged CYP in an outpatient mental health setting.	To get feedback from the graduate students on the programme.	Email survey	Categorising answers under the 4 competencies of the training.	5 (moderate)
D’Oosterlinck et al. (2009) [47]	Belgium	Allied	Staff working in residential facilities for children with emotional and behavioural disorders (*n* = 71*)	Empower the staff to handle conflict.	CYP with emotional and behavioural disorders who are in crisis in a therapeutic setting.	To evaluate whether the training was effective in empowering the staff members to handle the conflict.	Semi-structured questionnaire with open ended questions	Hermeneutic analysis with frequency coding and categorisation	3 (low)
Dababnah et al. (2019) [48]	Turkey	Allied	Teachers (n = 8)	To teach ASD knowledge, behavioural management, social support, and how to advocate for services and support in the community.	Syrian refugee CYP in Turkey, who have ASD, in addition to trauma.	To test the feasibility and acceptability of the training intervention.	Semi-structured interviews	Constant comparative method	9 (high)
Dame (2016) [49]	USA	Allied	Teachers (*n* = 9*)	Teach about childhood anxiety.	Anxious USA elementary school-aged children.	To evaluate changes in self-efficacy as result of the training, and to gauge perception of the training.	Focus groups	Classic transcript based analysis	8 (high)
David & Schiff (2018) [50]	Israel	Mental health	Clinicians practicing psychotherapy (*n* = 77*)	Training clinicians to use a bottom-up evidence based intervention for traumatised children and their families.	Young children with trauma.	To obtain information regarding whether, how, and where clinicians are using the intervention after training.	Open-ended questionnaire	Thematic analysis	8 (high)
Davies & Ray (2014) [51]	USA	Mental health	School psychologists (*n* = 19* at two-month follow-up; n = 18* at 1 year follow-up)	To increase awareness of TBI, better identify students with TBI, and improve education for students with TBI.	School-aged children with traumatic brain injury.	To evaluate the efficacy of a half-day traumatic brain injury training in school psychologists’ knowledge and skills.	Longitudinal survey	Content analysis	3 (low)
Donald (2015) [52]	USA	Allied	Residential care workers in psychiatric inpatient unit (n = 3*)	Child-teacher relationship training.	Can be used with general or clinical groups, aged 6–10 years.	To investigate training effects and experiences.	Semi-structured interviews	Thematic analysis	10 (high)
Drahota et al. (2014) [53]	USA	Mental health	Therapists (*n* = 13)	Train staff to deliver EBP programmes to reduce challenging behaviours in children with ASD.	CYP with ASD	To examine feasibility of implementation, from the perspectives of those receiving training on, and delivering, the EBPs.	Semi-structured interviews	A coding, consensus, and comparison methodology (an iterative approach rooted in grounded theory).	7 (moderate)
Dunsmuir et al. (2017) [54]	UK	Mental health	Tutors of school psychologists (n = 13)	Using problem based learning (PBL) with trainee school psychologists.	General school-aged population.	To evaluate strengths and weaknesses on PBL from different trainers.	Telephone survey	Thematic analysis	7 (moderate)
Eustache et al. (2017) [55]	Haiti	Allied	Teachers (*n* = 12*)	To prepare teachers to respond to student mental health needs.	A general school population.	To evaluate the feasibility and acceptability of this scope of training content and format of delivery, as well as its effectiveness in improving knowledge and attitudes relevant to school mental health.	Open-ended questionnaire and focus group discussions	Thematic analysis	8 (high)
Gonzalez et al. (2019) [56]	USA	Mental health	Trainers on school psychology training programmes (*n* = 327)	Teach about evidence-based assessment and intervention.	A general school-aged population	To conduct a more comprehensive and descriptive study of trainers’ instruction.	Online survey	Conventional content analysis	6 (moderate)
Grant et al. (2016) [57]	UK	Mixed	Medics, nurses, occupational therapists, radiographers, clinical and health psychologists (*n* = 31)	To teach about how having a very sick parent can impact CYP development, resilience, and family functioning.	CYP with a parent who has cancer.	To evaluate the implementation of training programme, and begin to establish its efficacy.	Semi structured open ended questionnaire	Framework analysis	7 (moderate)
Harris (2013) [58]	USA	Allied	PE teachers (n = 13*)	To improve confidence of PE to teachers include autistic children.	School-aged CYP with ASD.	To determine the effects of a 1 day in-service workshop on the self-efficacy and content knowledge of general physical educators.	Focus group	Reflexive analysis	9 (high)
Heyeres et al. (2018) [59]	Australia	Allied	Service manager, teachers, guidance counsellors, youth mentors (*n* = 21)	To support the wellbeing of indigenous students at boarding school, including mental health literacy and resilience training.	Australian Aboriginal children transitioning to boarding school at age 10–11 years.	To investigate whether the training influences the capacity of education staff to advocate for and support Indigenous student wellbeing.	Reflective group discussions, interviews	Thematic analysis	7 (moderate)
Jolivette et al. (2014) [60]	USA	Allied	Various school staff (n = 9*)	Train staff in a positive behavioural intervention, decreasing problem behaviours.	CYP between 7 and 17 years old, who have emotional and behavioural disorders.	To describe the training, and implementation effectiveness and fidelity.	Focus group	Constant comparative method	3 (low)
Jones & Howley (2010) [61]	UK	Allied	Local authority, teachers, SENcos (n = not stated)	To promote interactive skill building with children on the autism spectrum.	CYP with ASD	To investigate a system of training designed by a Local Education Authority support service.	Multi-method case study including semi-structured narrative approach interviews, and questionnaires.	Thematic analysis	7 (moderate)
Killick & Allen (2006) [62]	UK	Mixed	Staff on an adolescent psychiatric inpatient unit (nurses, psychologists, psychiatrists, social workers, teachers, family therapists) (*n* = 27*)	To manage aggressive and harmful behaviour in the adolescent psychiatric unit.	Psychiatric inpatient adolescents aged 11–18 years.	To evaluate effects of training - confidence increase, knowledge increase, good practice, staff satisfaction.	Survey	Grouping of comments - “informal analysis”	5 (moderate)
Lee (2016) [63]	UK	Allied	School pastoral support staff (n = 10*)	Increase knowledge of self-harm, identify risk factors, help CYP develop coping strategies, and develop a protocol for the school.	“Low-risk” self-harming secondary school aged CYP.	To explore each participant’s experiences of the workshop, and the meaning/psychological processes at work.	Semi-structured interviews	Interpretative phenomenological approach	6 (moderate)
Leventhal et al. (2018) [64]	India	Allied	Teachers (*n* = 24)	To help teachers improve mental health and other outcomes for youth in Low and Middle Income Countries.	A general school population of CYP, with a mean age of 13.5 years.	To describe key findings of the training and identify focus areas for the implementation going forward/scaling up.	Participatory action research - observations, focus group discussions, interviews, advisory groups.	Thematic analysis	2 (low)
Lusk et al. (2018) [65]	USA	Mental health	Psychiatric mental health nurse practitioner students (*n* = 107*)	To teach child/adolescent CBT to those on graduate nursing programmes.	CYP with mental health problems.	To evaluate the feasibility and acceptability of the model for advanced practice PMH students.	Open ended questionnaire	Thematic analysis	4 (moderate)
Manassis et al. (2009) [66]	Canada	Mental health	Practitioners from community mental health agencies (*n* = 22*)	To teach cognitive behavioural therapy for use with CYP.	CYP with internalising disorders	To evaluate the training	Interviews	Thematic analysis	5 (moderate)
Manning et al. (2017) [67]	UK	Allied	Children’s nurses (n = 8*)	Understand self-harm, effective communication with admitted CYP, assessing risk.	CYP who have self-harmed.	To determine the impact of the intervention on the knowledge, attitudes, confidence and behavioural intention of the staff, and to explore the perceived impact, suitability and usefulness of the intervention.	Semi-structured interviews	Thematic analysis	7 (moderate)
McAllister et al. (2019) [68]	Australia	Allied	Teachers, guidance officers, school nurses, indigenous school officer, chaplain, youth worker (n = 27*)	To develop knowledge and understanding of best practice in youth mental health promotion and to increase confidence in delivering the programme.	A general school population of Australian secondary school age CYP.	To report the results of the training.	Open-ended questionnaire	Thematic analysis	7 (moderate)
Omigbodun et al. (2007) [69]	Nigeria	Mixed	Multidisciplinary health professionals (nurses, doctors, psychologists, and community health workers) (*n* = 38*)	To provide a basic CAMH course.	Clinical population of CYP	To develop and evaluate the basic course with a multidisciplinary audience, to inform future training.	Open-ended questionnaire	Thematic analysis	6 (moderate)
Post et al. (2020) [70]	USA	Allied	Kindergarten teachers (n = 4)	Child-teacher relationship training.	USA kindergarten aged children.	Report findings of training in terms of teachers’ experiences.	Semi-structured interviews	Thematic analysis	6 (moderate)
Sherwin (2014) [71]	USA	Allied	Para-educators (n = 4)	Assist working with autistic children.	CYP with ASD.	To determine how para-educators respond to the training.	Interviews, questionnaire, focus groups	Organising into themes and building a theoretical model	9 (high)
Srivastava et al. (2015) [72]	India	Allied	Primary school teachers (*n* = 79*)	Improve attitudes and knowledge of special educational needs.	Primary school aged children with SEND.	To implement a teacher training program and evaluate its effects and appropriateness.	Open ended questionnaire	Thematic analysis	5 (moderate)
Suldo et al. (2010) [73]	USA	Mental health	School psychologists (*n* = 41*)	To improve knowledge and confidence with regards to suicide prevention for CYP.	A general school population, aged 5–18 years.	To evaluate the professional development intervention on youth suicide.	Open-ended questionnaire	Thematic analysis	5 (moderate)
Tchernegovski et al. (2015) [74]	Australia	Mental health	Mental health clinicians (nurses, social workers, psychologists) (n = 8*)	To provide clinicians with skills to empower parents with mental illness to support their family.	CYP of parents with a mental illness.	To examine clinicians’ views on the acceptability of the resource, and assesses its effectiveness.	Post-training interview	Thematic content analysis	7 (moderate)
Tilahun et al. (2017) [75]	Ethiopia	Allied	Community health workers (n = 11*)	A general CYP mental health course for community health workers.	CYP with mental health problems.	To examine training needs and perspectives in relation to providing child mental health care in rural Ethiopia.	Interviews	Framework analysis	8 (high)
Wu et al. (2019) [24]	Canada	Allied	Teachers, principals, plus one educational assistant (*n* = 23)	Train teachers to deliver PAX-GBG - a classroom-delivered mental health promotion intervention.	A general school population, up to 17 years old.	To gain a greater understanding of how the training was viewed by school personnel, in order to improve implementation in remote/indigenous communities.	Semi-structured interviews	Line-by-line analysis, grouping into categories, then themes	7 (moderate)

### Quality appraisal results

Using the JBI Critical Appraisal Checklist for Qualitative Research [34], each paper was scored out of 10 for quality. Each paper was then categorised into the ranks “low” (scores of 0–3); “moderate” (scores of 4–7); and “high” (scores of 8–10). 10% (*n* = 4) of the 39 papers were rated as low quality, 64% (*n* = 25) were rated as moderate quality, and 26% (*n* = 10) were rated as high quality. The precise score and rank assigned to each paper can be found in Table 2. Dissertations and theses tended to score higher, presumably owing to a more generous word count than peer-reviewed journal articles.

No papers were excluded from the synthesis on the basis of this appraisal. They key reason for that decision was the observation that certain items almost universally scored poorly. For example, only six of the 39 papers stated a philosophical perspective, and only five gave an indication of the researchers’ cultural or theoretical position. Other studies have found a similar pattern [76]. An explanation for this could be that studies in the healthcare field are often guided by pragmatic rather than philosophical or theoretical concerns [76]. Failing to report such standpoints does not necessarily undermine the utility of the study’s findings; however, the remainder of the items, such as declaring the study’s ethical approval, were deemed important quality indicators. Consequently, the results of the critical appraisal were not discounted entirely, and were considered when deciding how to organise, and proportionally draw from, the results of the synthesis.

### Level of credibility

The meta-aggregation method stipulates that a level of credibility should be assigned to each finding [34]. Consequently, after each finding was extracted, a supporting verbatim quotation was sought. Based on these illustrative quotations, the findings were categorised as “unequivocal”, meaning that the drawn conclusion reflects, beyond reasonable doubt, the views of the participant, or “equivocal”, meaning that although an association between the illustration and the finding can be deduced, the association is tenuous, or open to interpretation [34]. As the guidance suggests, findings rated unequivocal and equivocal were given equal recognition in the current synthesis. However, findings where a supporting quotation was not available were rated “unsupported”, and were consequently excluded from analysis. The process of assigning credibility ensures that participant voices are adequately represented by the authors’ interpretations, and that these interpretations are made transparent [34].

### Synthesised findings

Two hundred thirty raw findings were extracted from the 39 papers. When those rated “unsupported” in terms of credibility were removed, 182 raw findings remained. Then began the process of condensing and synthesising. As discussed earlier, longer findings were broken down and assigned more than one condensed finding, resulting in 219 condensed findings. These were summarised further into 47 sub-categories, 19 categories, and finally 5 synthesis statements. Twelve of the 39 studies included barriers and facilitators relating solely to the training process itself, with 6 dedicated to discussing the implementation of training in the workplace. Twenty-one studies discussed a mixture of both factors. Before synthesis, the raw findings were divided based on whether they related to training or implementation. The synthesis consequently produced two synthesis statements relating to the training process, and three relating to implementation. Three factors were taken into account, equally and in combination, when deciding how to order and present the data below. Priority and emphasis has been given to synthesis statements, categories, and sub-categories that a) score highest for quality; b) represent the greatest number of papers; and c) best represent both mental health and allied professionals. This means that within each overarching synthesis statement group (“training process” and “implementation”), the synthesis statements and categories will be discussed, and displayed in the accompanying figures, in order of strength based upon these three considerations.

#### Training process – synthesis statement 1: support

**Fig. 3:**
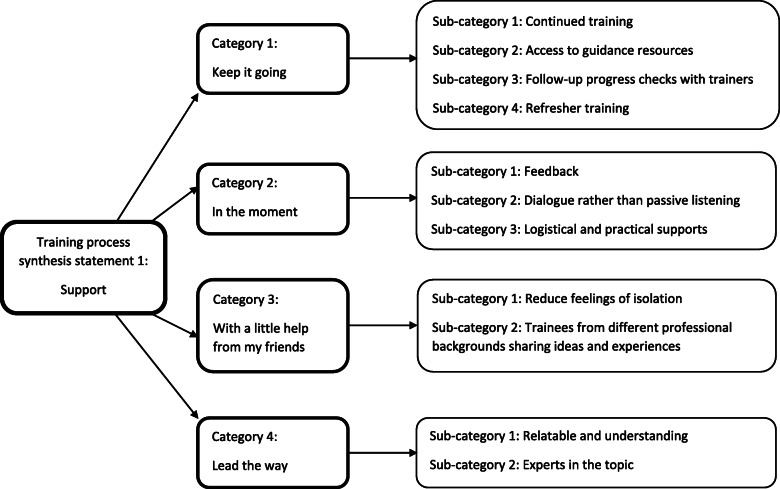
Training process – synthesis statement 1: Support

Professionals identified support as a vital part of the training process. This statement reflects the synthesis of four categories (Fig. 3*).*

##### Category 1: keep it going

The strongest category of findings in this synthesis statement was the idea that support should continue beyond the duration of the training programme. The desire for continued or additional training was frequently mentioned. Further training could build on already acquired knowledge or skills [55], or help imbed the knowledge into practice by maintaining familiarity and enthusiasm [57]. Indeed, providing follow-up training may maximise the future gains of the training in terms of future sustainability [44]. “Refresher training” could be offered, taking place on a regular, scheduled basis [69], so that trainees do not forget what they have learned [57] or lose confidence. The latter is a concern when training is used irregularly [62], or when many conflicting demands are present within the workplace [52].

Having access to guidance resources such as manuals or handbooks [53, 74], was reported as a valuable supportive tool, along with shared learning resources [56], or practical documents to use with clients [74]. Participants were reassured by having materials to refer to, and monitor their own usage of the training [52].

Ongoing dialogue with trainers would also have been welcomed, to talk through progress, acquire feedback [74], and to feel supported in their efforts to implement their training efficiently [45]. Routine progress checks should therefore be offered [59], plus a recourse for trainees to informally connect with trainers [53, 71].

##### Category 2: in the moment

Support should also be provided whilst training is taking place. Feedback should be given, and it is important that this feedback is personalised. This sense of individual guidance can boost skill development [53], with timely constructive criticism viewed as helpful [52]. Live supervision with immediate feedback during practical training is seen as a comforting support, and this method of learning is preferable to watching recorded sessions [70].

Linked to the latter observation, training sessions should allow open discussion between trainers and trainees: preferred over a lecture format [39, 58]. Trainees appreciate being listened to [58], with sufficient time spent within a group format to allow time for this [57].

Logistical and practical supports should also be taken into consideration. Training should be held in a suitable venue. As an example, trainees found it difficult to hear in a large room [24]. Training should ideally be free to undertake [74], with financial support offered if travel is necessary [55].

##### Category 3: with a little help from my friends

The importance of peer support was frequently highlighted. Training that facilitates interaction with peers, whether face-to-face or online, can reduce feelings of isolation [41]; with one participant describing the bonds they formed as almost familial [48]. Building a support network that may have been missing before [41] can be especially beneficial in terms of normalising the difficult emotions associated with certain professions [57], creating the sense that they are “all in this together” ([39], p. 203).

Sharing ideas with professionals from a wide range of backgrounds was often reported as helpful. Learning is facilitated [65] by allowing trainees to hear others’ experiences and take away new insights and ideas that can then be applied to their own practice [46]. It also provides valuable experience in working and collaborating with diverse teams of people [54].

##### Category 4: Lead the way

This category grouped findings pertaining to the ideal personal qualities of trainers. Firstly, trainers should be aware of their trainee audience, possessing insight into their roles and circumstances. They can then provide tailored, specific guidance [24]. They should also be mindful of the varying levels of background knowledge and experience the trainees might have, especially when training an assortment of professionals. Nurses and teachers, for example, are likely to approach the training from very different perspectives [68]. This high level of understanding should make the trainer relatable and approachable should trainees wish to ask questions [58].

Trainers should also be experts in the topic they are delivering: learning is best facilitated by a high quality trainer [39, 68] who is knowledgeable and passionate about the subject matter [43]. Indeed, a lack of expertise can limit the potential of the training, influencing its subsequent transfer to practice [56].

#### Training process – synthesis statement 2: training content, design, and planning

**Fig. 4:**
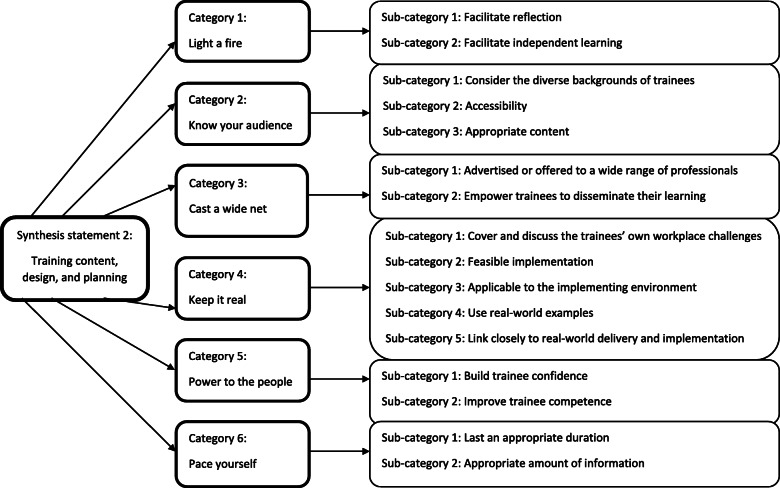
Training process – synthesis statement 2: Training content, design, and planning

Six categories were identified, encompassing the nature of the training programmes themselves (Fig. 4*)*. This synthesis statement provides recommendations for what training should ideally offer.

##### Category 1: light a fire

Training should act as a catalyst for further reflection and learning. It should be thought provoking [67] actively promoting introspection on one’s own practice [41]. Challenging one’s own preconceptions in this manner can build compassion towards clients [67]: crucial in a mental health context. Trainees were also encouraged view their role with a wider lens, in terms of how their work fits in with other services [41], how important their role is [39] or enlightening them as to the scope of the issues at hand [43].

Training should also provide a solid foundation for independent learning, helping trainees to become active in their own skill acquisition [54]. One way to promote this is by limiting the amount of information available at the outset of training, allowing space for reflection and mistakes [52]. Although time-consuming, this may be an effective method of knowledge consolidation.

##### Category 2: know your audience

Training should be suitable for its target group of trainees. Firstly, diversity should be borne in mind when designing training. The literature highlighted potential language barriers, especially if a significant proportion of trainees speak the language of instruction as a second language [75], and the importance of respecting cultural sensitivities [55]. At a professional level, if trainees come from a diverse range of professional backgrounds, it may be pertinent to provide separate, tailored training, ensuring that content is relevant and accessible by all [24].

Accessibility is an important concept to explore further. Training information should be easy to understand [39]; unclear language or jargon should be thoroughly explained [56]. Special attention should be given to supporting those using online or digital materials [74]. Varying activities is seen as positive, as it caters for a wider range of preferences and strengths [68], and scaffolding between training stages, so that skills build upon each other, is a helpful way of ensuring understanding [52].

Taking diversity and accessibility into consideration, a more general observation is that the training content itself should be appropriate. The level of difficulty should be appropriate, as it is frustrating for trainees when they are already familiar with the content [63]. Good communication should exist between trainers: their agreement on what needs to be covered [56] should ensure comprehensive yet relevant coverage [74].

##### Category 3: cast a wide net

Training should be offered to as wide a range of relevant professionals as possible, to ensure consistency of approach when dealing with CYP mental health [24]. For example, teachers attending one training programme suggested that it should be offered to other professional groups, such as the police, so that help can be sought anywhere [55]. Expanding training in this way can also promote cross-professional idea sharing [69]. In addition, raising awareness of CYP mental health training and interventions by educating the general public may further widen the impact of such programmes [69].

Dissemination of acquired knowledge to others through trainees is another important way to extend the reach of training. Training programmes should therefore empower trainees to confidently disseminate their knowledge [69], or train others back in their workplace [61].

##### Category 4: keep it real

Training should tie in closely with the reality of the environment in which it is due to be implemented. Trainees appreciated the chance to raise and discuss the specific challenges they were facing at work, which they valued more than studying pre-prepared examples [58] or theoretical overviews [42, 44]. Discussion of real-world issues can build empathy towards clients [39], especially when explored through multiple perspectives [43].

The workplace application of training should be as feasible as possible for maximum impact. Elements of training were seen as unhelpful if delivery simply would not work “on the ground”, due to, for example, time constraints [74]. Extra care should be taken with interventions that were designed in research settings, to ensure that they are adaptable to local realities [56]. Immediate practical application of skills leads to training being viewed positively, as well as maximising its effectiveness [41]. Additionally, training should be delivered at a pertinent time, for example at a suitable point in the school year [24], or when a particular issue is salient.

Learning should be directly relevant to practical implementation [68]: a lack of clarity on how to apply training is a clear barrier [24]. It follows that guiding trainees through the practical application of training, and how to overcome the possible dilemmas faced, would be appreciated [51].

##### Category 5: power to the people

Building staff confidence, knowledge, and competence can improve workplace capability [54]. Several studies reported that training improved trainee confidence [49, 67]. It can do this through reducing anxiety [57] and reassuring them that they are already doing well [61], ultimately building faith in their own abilities [41]. Training should also teach a solutions-focussed way of thinking that allows trainees to face challenges pragmatically [57].

##### Category 6: pace yourself

The pacing of training should be considered. Long sessions can be overwhelming [68], so regular breaks should be given to avoid fatigue [55]. The coverage of multiple topics, however, should be undertaken in longer or multiple sessions, so sufficient time can be given to each [69] building a fuller understanding [39].

#### Implementation – synthesis statement 1: contextual factors

**Fig. 5:**
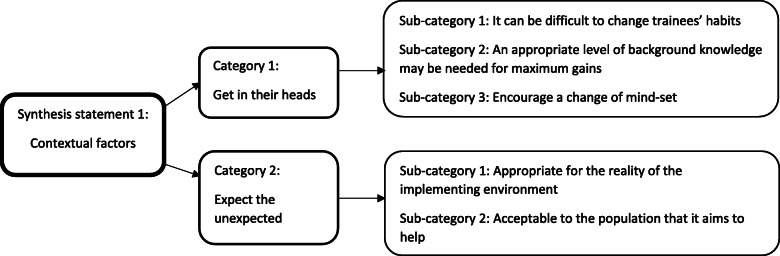
Implementation – synthesis statement 1: Contextual factors

This synthesis statement explores the barriers and facilitators underpinning successful workplace implementation of training. It comprises two categories (Fig. 5).

##### Category 1: get in their heads

Changing habits and mind-sets of trainees can be difficult. This can influence how seamlessly training becomes embedded into daily practice. Trainees are often used to carrying out their work in a particular way [52], and changes can be stressful for both staff and clients [71]. Training should therefore encourage a change of mind-set by actively challenging existing views and prejudices [67], and helping organisations to overwrite out-dated beliefs and practices [47]. Fortunately, one study suggested that reframing knowledge, (in this example, trainees were taught to imagine mental health as a continuum rather than as “health” versus “illness”), can happen quickly when this is facilitated effectively in training [41].

For successful implementation, familiarity with the trained intervention must reach a certain level, in order to meaningfully learn and apply the information. Using simple language, and thorough explanations of basic concepts, can aid their understanding [75]. For this reason, implementation often happens “bit by bit”, with gradual benefits [24].

##### Category 2: expect the unexpected

This category suggests that implementation should be flexible enough to use in real workplaces - contexts that are diverse and unpredictable by nature. CYP mental health training in particular must be flexible enough to apply to clients’ unique therapeutic needs [41]. If training fails to consider these diverse needs and contexts, it can be difficult to apply it [24, 47]. One study, for example, reported challenges with applying an intervention ill-suited for children with communication problems [48]. Another reported concerns about using aggression management with physically larger adolescents [62]. Additionally, implementations should be flexible enough to provide value to all targeted organisations, in terms of client age group [24] or special needs status [58].

Training should also consider the various ways of working and therapeutic styles of staff, perhaps suggesting multiple usage strategies [74]: providing such flexibility can improve acceptability. Cultural and religious beliefs held by certain populations may influence how receptive clients and their families are to treatments, and financial problems may also limit suitability [75]. Adapting the training content to suit the targeted population may alleviate these issues.

#### Implementation – synthesis statement 2: perceived value

**Fig. 6:**
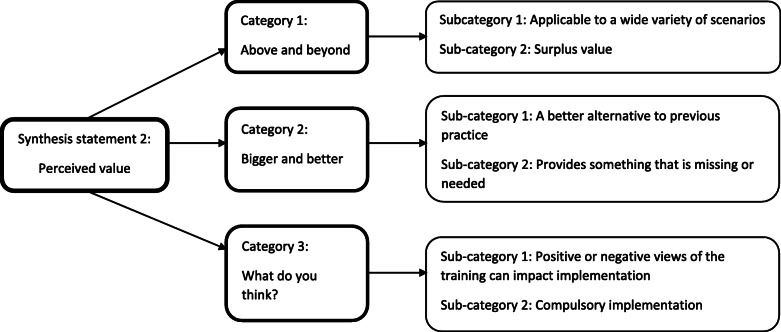
Implementation – synthesis statement 2: Perceived value

This statement consolidates three categories *(*Fig. 6*)* that discuss trainees’ attitudes towards the training, and how these can influence their ability to implement the skills at work.

##### Category 1: above and beyond

If trainees perceive that the skills they have learned can be applied widely to their work, successful implementation is more likely. For example, training can boost staff confidence when dealing with a wider range of clients than usual [41]. If staff members view the training as having wide value, they are also more likely to recommend it to others, increasing its reach [48].

The wide application of training feeds into the idea that training can sometimes provide the skills needed to work with a broader range of people or scenarios than it originally intended. Training that goes “above and beyond” in this manner can be said to have surplus value, and is generally viewed favourably by trainees. They appreciate being able to apply training to all CYP they encounter at work, not solely those the training was aimed at [48], and some reported gaining skills that could be applied to their family or community lives, as well as at work [47, 55].

##### Category 2: bigger and better

Trainees should view their learning as a strong alternative to systems or procedures that are already in practice. It should enable them to handle situations in a stronger, more effective way [70], providing feasible alternatives that are especially valued when current methods are viewed negatively. For example, staff in one study appreciated being taught alternative methods that helped them avoid restraining or medicating patients [42].

Training that provides something that is missing or needed in the workplace may result in better implementation. This perceived need can build enthusiasm, which may encourage implementation [52, 64]. Trainees commonly reported that training provided a structure to lean on, which they felt was missing previously. These structures can provide focus to therapy sessions, allowing them to target specific behaviours or issues [53]. This was greatly appreciated by those working in high-pressured, conflict-heavy situations [47].

##### Category 3: what do you think?

Related to the previous category, it was found that trainees’ attitudes towards the training were important. If the implementing organisation or workplace views the training as valuable, implementation will be more likely. One study reported that time, effort, and resources were dedicated to implementation because the training was well recognised, and perceived as valuable [61]. Another study reported that mandating implementation following training encourages change [74]. On the other side of the coin, hearing negative information about training programmes from colleagues can reduce receptiveness to both attending and implementing the training [24].

#### Implementation – synthesis statement 3: Organisational factors

**Fig. 7:**
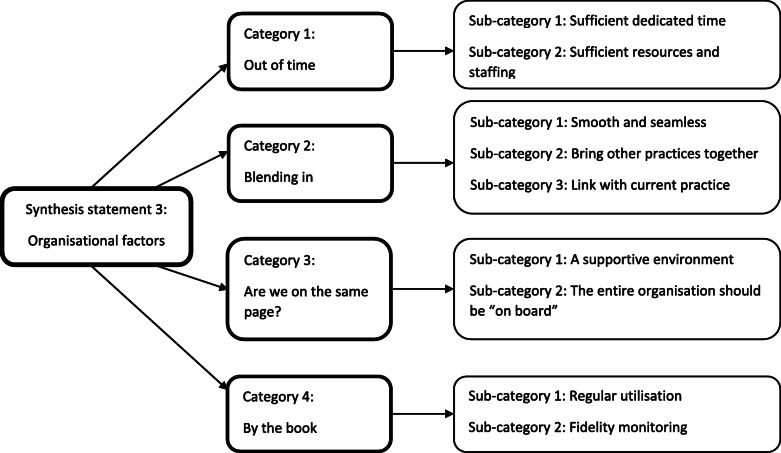
Implementation – synthesis statement 3: Organisational factors

The final synthesis statement relates to factors within the implementing organisation, which were suggested as influential to the success of application. It involved the synthesis of four categories *(*Fig. 7*).*

##### Category 1: out of time

Resource availability appears to be an important predictor of training implementation. A key resource is time, a lack of which can limit the sustainability of training gains [44]. Trainees reported challenges with finding time to utilise the training, struggling to embed it into a busy schedule with conflicting demands [42]. These demands often take priority, meaning that the training does not get used [47]. There are therefore several recommendations that warrant attention. The time constraints of each workplace should be considered to assess feasibility [38], namely how it will fit into schedules that are often rigid [56]. Logistical factors, such as time spent preparing rooms, should also be factored in [61], along with the fact that extra time may be needed at the beginning, to account for the concentration and precision needed to simultaneously learn and implement [48]. Whether an organisation has sufficient and appropriate physical resources, such as equipment and space, are also vital if training can be implemented to its full potential [48], along with staffing levels [47], and consideration of how implementation will fare in the face of absenteeism [64].

##### Category 2: blending in

Implementation should be as smooth and seamless as possible. New processes should not be clunky and cumbersome to implement, rather they should feel natural to use in the workplace [50]. Ideally, implementation should align well with the ideologies and attitudes that are held by organisations [54]: trainees are more likely to use skills if a large deviation in their way of thinking is not required. In addition, if trainees are able to see an overlap between the training and skills that they already use, the consistency is appreciated [53]. Indeed, a good training programme could help unify other learnings and sources [59] that previously seemed distinct and isolated [53].

##### Category 3: are we on the same page?

The idea of a cohesive workplace, where the entire organisation is “on board” with implementation, was evidenced as important. Trainees mentioned that implementation was simplified because all staff in their organisation were trained. The way they interacted with CYP was therefore consistent [24]. This sentiment was echoed by a trainee in another study, who wished that all staff members in their workplace were at least aware of the intervention [52]. Explaining, or justifying the use of, new skills can prove challenging when others are not aware of them [52]. In summary, if everybody is “on the same page” ([60], p. 76), implementation goals are more likely to be met.

It follows that a supportive workplace is the ideal environment for implementation. Trainees mentioned that the support of senior staff in their organisation was of vital importance [24], and although the challenges of implementation can be overcome by a small number of motivated individuals, it can be extremely difficult without helpful, supportive colleagues [64].

##### Category 4: by the book

This category combined a small number of findings relating to implementation fidelity. For maximum gain, implementation should adhere as closely as possible to the model stipulated within training guidance. To ensure high fidelity, monitoring may be necessary. This could be facilitated by additional trainer support, especially when trainees express concern about whether they are implementing skills correctly [57]. Regular use of training is also vital. If trainees do not have an opportunity to use the new skills, for whatever reason, their confidence will decline, resulting in a further reduction of use [62].

## Discussion

### Summary

This systematic review and qualitative meta-aggregation sought to investigate the experiences of mental health and allied professionals, who undertook training relating to the mental health of CYP. Specifically, it aimed to identify the barriers and facilitators that these professionals reported as having hindered or helped them during the training process, and in the subsequent implementation of the trained skills back in the workplace. To our knowledge, this is the first review of training experiences that focuses solely on those who work with CYP, and that synthesises literature pertaining to both mental health *and* allied professionals. It is hoped that the review will provide accessible and organised guidance for those designing and delivering training, as well as for leaders within implementing workplaces who desire to make the training that their employees receive as effective as possible.

Literature that qualitatively explored these barriers and facilitators was identified systematically, and the resulting 39 papers were synthesised using meta-aggregation: a method used to develop practical, directive action points that are a synthesised version of the authors’ original intended meanings [34]. Two synthesis statements relating to the training process, and three relating to workplace implementation, were identified. Support from peers and trainers, both during and after training were seen as vital for maximisation of training gains, as were several qualities of the training itself, such as reach, pacing, and suitability. The success of implementation in the workplace depended firstly upon contextual factors, including the prior knowledge and habits of trainees, and the applicability of new skills to unpredictable workplace scenarios. Secondly, perceived value was seen as important, in terms of both broad worth, and whether it provided a better alternative to current practice. Finally, qualities within the organisation itself, such as available resources, workplace cohesiveness, and the potential for implementation fidelity, were notable.

At first glance, the findings of the review are unlikely to cause a great deal of surprise, and may be seen as “common-sense” principles. However, since the review collates the voices of those very professionals that it aims to produce guidance for, this review provides strong evidence-based backing to principles that, as evidenced in the literature, resonate widely. It ties widely appreciated qualities into a coherent and organised framework, giving structure for the reader, and more importantly, providing an accessible resource for those involved in training delivery or implementation.

### Guidance for use

Some of the recommendations, for instance that feelings of support and empowerment should emanate from training, can be applied almost universally to any training or implementation scenario. However, it should also be pointed out that not all recommendations are suitable for every training programme or organisation. It is expected, therefore, that those using the review will choose recommendations that are suitable within their own context. For example, whilst a training programme that allows flexible implementation could be extremely useful when broad application to a large range of CYP is desired, for training that is very specific by nature, perhaps only destined for use within a niche area of mental health, this is not a relevant recommendation. This idea is also evidenced by the fact that some categories seem to contradict one another. The category “get in their heads” recommends that if successful implementation is to occur, training should challenge trainees’ existing views by reframing knowledge, and challenging out-dated beliefs. Conversely, the category “blending in” suggests that a large deviation in thinking should not be required. Although these findings appear to oppose one another, users can strike their own balance between the two, again depending on their own contexts and requirements. Indeed, “get in their heads” points out that implementation often happens gradually as mind-sets adapt, which could be borne in mind as an idea that bridges the gap between the two recommendations. Whilst change is important, perhaps it does not need to happen instantly. Another similar example is the suggestion that training should be able to be implemented flexibly (“expect the unexpected”) but also with fidelity (“by the book”). Whilst some empirical studies have found that implementation fidelity does predict its long-term sustainability, (e.g. [77]), over-rigid programme adherence might be detrimental [78]. Whilst a training intervention should remain recognisable in spite of any adaptations, inflexibility can result in a close-minded culture that is reluctant to integrate better practices [79]. This again suggests the importance of balance, and of contemplating the intricacies of each training situation when considering the recommendations in this review.

Some categories also appear to cover similar content. “Expect the unexpected” and “above and beyond”, for example, both refer to the wide applicability of training to multiple scenarios, despite falling under separate synthesis statements. This owes to the fact that the findings of “expect the unexpected” relate to the context of the implementing workplace and the influencing factors within it, whereas those forming “above and beyond” are discussions of participants’ perceptions of the training’s personal value. Thus, they were categorised and synthesised according to how the participants framed their observations.

### Strengths and limitations

As Fig. 2 shows*,* this is a comprehensive review. Over 12,000 titles and abstracts were screened, which were eventually narrowed down to 39 eligible papers. Regarding the state of the field, we reported that only 10 of these papers were classified as “high quality” using the JBI Critical Appraisal Checklist for Qualitative Research [34], however 25 were marked as “moderate quality”. As discussed, the varying relevance of the items in the appraisal tool led to the inclusion of all papers. However, the scores were utilised alongside volume of evidence, and professional representation when constructing the results section, in order to organise and emphasise the findings. This led to the development of strong, evidence-based principles, presented based upon an intersection of quality, frequency, utility, and representativeness.

Meta-aggregation is occasionally discounted as a valid method of meta-synthesis. A critical paper accuses the method of turning “rich descriptions into thin abstractions” ([80], p. 7), and noted a lack of re-interpretation and generation of new theories. However, given that the aim of this review was to generate practical statements to guide change, a reliable collation of study findings, presented as intended [34], was undoubtedly useful. This aim negates the need for new, overarching interpretations to be made.

Meta-aggregation is the only qualitative meta-synthesis method that aligns with the philosophical approach of pragmatism [36]. Akin to all qualitative synthesis methods, it can come under scrutiny for not producing entirely replicable results, and although meta-aggregation is the most practical, structured form of qualitative meta-synthesis [36], the process still involves a level of subjectivity that would not occur in quantitative work. Perhaps a different set of synthesis statements and categories may have been produced had the process been conducted by a different set of authors. Despite this, we tend to agree with the stance that although an identical level of rigour should be applied to both qualitative and quantitative syntheses, transparency should be the ultimate goal when presenting the former, rather than reproducibility [81]. The essence of qualitative synthesis lies in “making structured judgements” ([81], p. 258), and providing that the framework underpinning the authors’ thought processes is made transparent, the judgement outcomes do not need to be replicable. We hope that this has been achieved in this review.

## Conclusions

Five synthesis statements were produced using qualitative meta-aggregation. Two of these related to the process of training and its delivery, and three related to the implementation of the training back in the workplace. The synthesis statements, and underlying categories, provide practical recommendations for those designing, delivering, or implementing CYP mental health training, with a range of both mental health, and allied, professionals. They can be used to improve training content and delivery, and to maximise gains during implementation. The review provides a strong evidence-based foundation to “common-sense” principles, drawing them into a coherent and organised framework using a synthesis method grounded in pragmatism.

## Supplementary Information

**Additional file 1.**

## Data Availability

PROSPERO reference is included in the manuscript. An example search strategy is included as supplementary material.

## Abbreviations

A&E: “Accident and Emergency” (ER/emergency department)
CAMHS: Child and Adolescent Mental Health Services
CYP: Children and young people
PRISMA: Preferred reporting items for systematic reviews and meta-analyses

## References

[1] NHS Digital. Mental Health of Children and Young People in England, 2020: Wave 1 follow up to the 2017 survey. Government Statistical Service. 2020. Available from https://digital.nhs.uk/data-and-information/publications/statistical/mental-health-of-children-and-young-people-in-england/2020-wave-1-follow-up#. Accessed 27 Nov 2020.

[2] World Health Organisation (2020). Improving the mental and brain health of children and adolescents.

[3] Jones PB (2013). Adult mental health disorders and their age at onset. BJPsych..

[4] Trotta A, Arseneault L, Caspi A, Moffitt TE, Danese A, Pariante C, Fisher HL (2020). Mental health and functional outcomes in young adulthood of children with psychotic symptoms: a longitudinal cohort study. Schizophr Bull.

[5] Aebi M, Giger J, Plattner B, Metzke CW, Steinhausen HC (2014). Problem coping skills, psychosocial adversities and mental health problems in children and adolescents as predictors of criminal outcomes in young adulthood. Eur Child Adolesc Psychiatry.

[6] Naicker K, Galambos NL, Zeng Y, Senthilselvan A, Colman I (2013). Social, demographic, and health outcomes in the 10 years following adolescent depression. J Adolesc Health.

[7] Centre for Mental Health (2010). The economic and social costs of mental health problems in 2009/10.

[8] Belfer ML (2008). Child and adolescent mental disorders: the magnitude of the problem across the globe. J Child Psychol Psychiatry.

[9] Edwards R, Williams R, Dogra N, O'Reilly M, Vostanis P (2008). Facilitating and limiting factors of training available to staff of specialist CAMHS. J Ment Health Train Educ Pract.

[10] NHS England. Implementing the five year forward view for mental health. NHS England 2015. Available from https://www.england.nhs.uk/wp-content/uploads/2016/07/fyfv-mh.pdf. Accessed 24 Nov 2020.

[11] Neufeld SA, Dunn VJ, Jones PB, Croudace TJ, Goodyer IM (2017). Reduction in adolescent depression after contact with mental health services: a longitudinal cohort study in the UK. Lancet Psychiatry.

[12] Neufeld SA, Jones PB, Goodyer IM (2017). Child and adolescent mental health services: longitudinal data sheds light on current policy for psychological interventions in the community. J Public Ment Health.

[13] Wolpert M, Harris R, Hodges S, Fuggle P, James R, Wiener A, McKenna C, Law D, York A, Jones M, Fonagy P, Fleming I, Munk S (2016). THRIVE Elaborated.

[14] Smith J, Kyle RG, Daniel B, Hubbard G (2018). Patterns of referral and waiting times for specialist child and adolescent mental health services. Child Adolesc Ment Health.

[15] Department of Health (2015). Future in Mind: Promoting, protecting, and improving our children and young people’s mental health and wellbeing.

[16] Duong MT, Bruns EJ, Lee K, Cox S, Coifman J, Mayworm A, Lyon AR (2020). Rates of mental health service utilization by children and adolescents in schools and other common service settings: a systematic review and meta-analysis. Admin Pol Ment Health.

[17] Ford T, Hamilton H, Goodman R, Meltzer H (2005). Service contacts among the children participating in the British child and adolescent mental health surveys. Child Adolesc Ment Health..

[18] O’Reilly M, Adams S, Whiteman N, Hughes J, Reilly P, Dogra N (2018). Whose responsibility is adolescent’s mental health in the UK? Perspectives of key stakeholders. School Ment Health..

[19] Hinrichs S, Owens M, Dunn V, Goodyer I. General practitioner experience and perception of Child and Adolescent Mental Health Services (CAMHS) care pathways: a multimethod research study. BMJ Open. 2012;2(6). 10.1136/bmjopen-2012-001573. 10.1136/bmjopen-2012-001573 PMC3533003 23148343

[20] Kerasidou A, Kingori P (2019). Austerity measures and the transforming role of a&E professionals in a weakening welfare system. Plos One.

[21] Community Practitioner (2016). Children’s mental illness stretching a&E services. Community Pract.

[22] Timson D, Priest H, Clark-Carter D (2012). Adolescents who self-harm: professional staff knowledge, attitudes and training needs. J Adolesc.

[23] Wolpert M, Harris R, Hodges S, Fuggle P, James R, Wiener A, McKenna C, Law D, York A, Jones M, Fonagy P, Fleming I, Munk S (2019). THRIVE framework for system change.

[24] Wu YQ, Chartier M, Ly G, Phanlouvong A, Thomas S, Weenusk J (2019). Qualitative case study investigating PAX-good behaviour game in first nations communities: insight into school personnel's perspectives in implementing a whole school approach to promote youth mental health. BMJ Open.

[25] Wei Y, McGrath PJ, Hayden J, Kutcher S (2015). Mental health literacy measures evaluating knowledge, attitudes and help-seeking: a scoping review. BMC Psychiatry.

[26] Kutcher S, Wei Y, Coniglio C (2016). Mental health literacy: past, present, and future. Can J Psychiatr.

[27] Haggerty D, Carlson JS, McNall M, Lee K, Williams S (2019). Exploring youth mental health first aider training outcomes by workforce affiliation: a survey of project AWARE participants. School Ment Health.

[28] Lempp T, Heinzel-Gutenbrunner M, Bachmann C (2016). Child and adolescent psychiatry: which knowledge and skills do primary care physicians need to have? A survey in general practitioners and paediatricians. Eur Child Adolesc Psychiatry..

[29] Beidas RS, Kendall PC, editors. Dissemination and implementation of evidence-based practices in child and adolescent mental health. New York: Oxford University Press; 2014.

[30] Scantlebury A, Parker A, Booth A, McDaid C, Mitchell N (2018). Implementing mental health training programmes for non-mental health trained professionals: a qualitative synthesis. Plos One.

[31] World Health Organisation (2020). Adolescent health in the South-East Asia Region.

[32] Sawyer SM, Azzopardi PS, Wickremarathne D, Patton GC (2018). The age of adolescence. Lancet Child Adolesc Health.

[33] Ayorinde AA, Williams I, Mannion R, Song F, Skrybant M, Lilford RJ, Chen YF (2020). Assessment of publication bias and outcome reporting bias in systematic reviews of health services and delivery research: a meta-epidemiological study. Plos One.

[34] Lockwood C, Munn Z, Porritt K (2015). Qualitative research synthesis: methodological guidance for systematic reviewers utilizing meta-aggregation. Int J Evid Based Healthc..

[35] Boland A, Cherry M, Dickson R (2017). Doing a systematic review: a student’s guide.

[36] Hannes K, Lockwood C (2011). Pragmatism as the philosophical foundation for the Joanna Briggs meta-aggregative approach to qualitative evidence synthesis. J Adv Nurs.

[37] Johnson SF, Woodgate RL (2017). Qualitative research in teen experiences living with food-induced anaphylaxis: a meta-aggregation. J Adv Nurs.

[38] Adelman AS (2014). Structured observation as a professional development tool to prepare teachers for observing social responsiveness in preschool students with autism spectrum disorder (doctoral dissertation).

[39] Askell-Williams H, Murray-Harvey R (2016). Sustainable professional learning for early childhood educators: lessons from an Australia-wide mental health promotion initiative. J Early Child Res.

[40] Bassilios B, Nicholas A, Ftanou M, Fletcher J, Reifels L, King K, Machlin A, Pirkis J (2017). Implementing a primary mental health service for children: administrator and provider perspectives. J Child Fam Stud.

[41] Bazyk S, Demirjian L, LaGuardia T, Thompson-Repas K, Conway C, Michaud P. Building Capacity of Occupational Therapy Practitioners to Address the Mental Health Needs of Children and Youth: A Mixed-Methods Study of Knowledge Translation. Am J Occup Ther. 2015;69(6):6906180060p1–690618006010. doi: 10.5014/ajot.2015.019182 10.5014/ajot.2015.019182 PMC4643378 26565099

[42] Blackburn J, McKenna B, Jackson B, Hitch D, Benitez J, McLennan C, et al. Educating mental health clinicians about sensory modulation to enhance clinical practice in a youth acute inpatient mental health unit: a feasibility study. Issues in mental health nursing. 2016;37(7):517-25. Issues Ment Health Nurs. 10.1080/01612840.2016.1184361. 10.1080/01612840.2016.1184361 27253182

[43] Bond E, Dogaru C (2019). An evaluation of an inter-disciplinary training Programme for professionals to support children and their families who have been sexually abused online. Br J Soc Work.

[44] Bryson SA, Ostmeyer KF (2014). Increasing the effectiveness of community mental health center social skills groups for children with autism Spectrum disorder: a training and consultation example. Admin Pol Ment Health.

[45] Christie G, Black S, Dunbar L, Pulford J, Wheeler A (2013). Attitudes, skills and knowledge change in child and adolescent mental health workers following AOD screening and brief intervention training. Int J Ment Health Addict.

[46] Coiro MJ, Kotchick BA, Preis J (2016). Youth social skills groups: a training platform for promoting graduate clinician interprofessional competence. J Interprof Educ Pract.

[47] D'Oosterlinck F, Soenen B, Goethals I, Vandevelde S, Broekaert E (2009). Perceptions of staff members on the implementation of conflict management strategies in educational and therapeutic environments for children and youths with emotional and Behavioural disorders. Ther Communities.

[48] Dababnah S, Habayeb S, Bear BJ, Hussein D (2019). Feasibility of a trauma-informed parent–teacher cooperative training program for Syrian refugee children with autism. Autism..

[49] Dame SL (2016). Training elementary teachers about childhood anxiety: perception and self-efficacy (doctoral dissertation).

[50] David P, Schiff M (2018). Initial clinician reports of the bottom-up dissemination of an evidence-based intervention for early childhood trauma. Child Youth Care Forum.

[51] Davies SC, Ray AM (2014). Traumatic brain injury: the efficacy of a half-day training for school psychologists. Contemp Sch Psychol.

[52] Donald E (2015). The effects of child teacher relationship training (CTRT) on residential care workers: a mixed methods study (doctoral dissertation).

[53] Drahota A, Stadnick N, Brookman-frazee L (2014). Therapist perspectives on training in a package of evidence-based practice strategies for children with autism Spectrum disorders served in community mental health clinics. Admin Pol Ment Health.

[54] Dunsmuir S, Frederickson N, Lang J. Meeting current challenges in school psychology training: The role of problem-based learning. School Psych Rev. 2017;46(4):395–407. doi:10.17105/SPR-2016-0017.V46-4

[55] Eustache E, Gerbasi ME, Smith Fawzi MC, Fils-Aime JR, Severe J, Raviola GJ (2017). Mental health training for secondary school teachers in Haiti: a mixed methods, prospective, formative research study of feasibility, acceptability, and effectiveness in knowledge acquisition. Glob Ment Health.

[56] Gonzalez JE, Stoiber KC, Clayton RJ, Keller-Margulis M, Reddy LA, Forman SG. A qualitative analysis of school psychology trainers’ perspectives on evidence-based practices. Int J Sch Educ Psychol. 2019:1–16. 10.1080/21683603.2019.1668317.

[57] Grant L, Sangha A, Lister S, Wiseman T (2016). Cancer and the family: assessment, communication and brief interventions—the development of an educational programme for healthcare professionals when a parent has cancer. BMJ Support Palliat Care.

[58] Harris N (2013). The effects of a one day in-service workshop on the self-efficacy of physical educators to include students with autism into the general physical education setting (doctoral dissertation).

[59] Heyeres M, McCalman J, Langham E, Bainbridge R, Redman-Maclaren M, Britton A (2019). Strengthening the capacity of education staff to support the wellbeing of indigenous students in boarding schools: a participatory action research study. Aust J Indig Educ.

[60] Jolivette K, Patterson DP, Swoszowski NC, McDaniel SC, Kennedy C, Ennis RP (2014). School-wide positive behavioral interventions and supports in a residential School for Students with Emotional and Behavioral Disorders: first years of implementation and maintenance follow-up focus groups. Resid Treat Child Youth.

[61] Jones K, Howley M (2010). An investigation into an interaction programme for children on the autism spectrum: outcomes for children, perceptions of schools and a model for training. J Res Spec Educ Needs.

[62] Killick S, Allen D (2006). Training staff in an adolescent inpatient psychiatric unit in positive approaches to managing aggressive and harmful behaviour: does it improve confidence and knowledge?. Child Care Pract.

[63] Lee F (2016). Self-harm training in secondary schools: an educational psychology intervention using interpretative phenomenological analysis. Educ Child Psychol.

[64] Leventhal KS, Andrew G, Collins CS, DeMaria L, Singh HS, Leventhal S (2018). Training school teachers to promote mental and social well-being in low and middle income countries: lessons to facilitate scale-up from a participatory action research trial of youth first in India. Int J Emot Educ.

[65] Lusk P, Hart Abney BG, Melnyk BM (2018). A successful model for clinical training in child/adolescent cognitive behavior therapy for graduate psychiatric advanced practice nursing students. J Am Psychiatr Nurses Assoc.

[66] Manassis K, Ickowicz A, Picard E, Antle B, McNeill T, Chahauver A (2009). An innovative child CBT training model for community mental health practitioners in Ontario. Acad Psychiatry.

[67] Manning JC, Carter T, Latif A, Horsley A, Cooper J, Armstrong M, et al. ‘Our Care through Our Eyes’. Impact of a co-produced digital educational programme on nurses’ knowledge, confidence and attitudes in providing care for children and young people who have self-harmed: a mixed-methods study in the UK. BMJ Open. 2017;7(4):1. doi:10.1136/bmjopen-2016-014750 10.1136/bmjopen-2016-014750 PMC5623397 28473515

[68] McAllister M, Knight BA, Handley C, Withyman C, Dawkins J, Hasking P (2019). Evaluation of a professional development experience designed to equip school support staff with skills to facilitate youth mental health promotion. Contemp Nurse.

[69] Omigbodun O, Bella T, Dogra N, Simoyan O (2007). Training health professionals for child and adolescent mental health care in Nigeria: a qualitative analysis. Child Adolesc Ment Health..

[70] Post PB, Grybush AL, Elmadani A, Lockhart CE (2020). Fostering resilience in classrooms through child-teacher relationship training. Int J Play Ther.

[71] Sherwin M (2014). An investigation into best practices in training Para-educators of students with autism (doctoral dissertation).

[72] Srivastava M, de Boer AA, Pijl SJ (2015). Know how to teach me … evaluating the effects of an in-service training program for regular school teachers toward inclusive education. Int J Sch Educ Psychol.

[73] Suldo S, Loker T, Friedrich A, Sundman A, Cunningham J, Saari B, et al. Improving School Psychologists’ Knowledge and Confidence Pertinent to Suicide Prevention Through Professional Development. J Appl Sch Psychol. 2010;26(3):177–97. doi:10.1080/15377903.2010.495919.

[74] Tchernegovski P, Reupert A, Maybery D (2015). “Let's talk about children”: a pilot evaluation of an e-learning resource for mental health clinicians. Clin Psychol.

[75] Tilahun D, Hanlon C, Araya M, Davey B, Hoekstra RA, Fekadu A (2017). Training needs and perspectives of community health workers in relation to integrating child mental health care into primary health care in a rural setting in sub-Saharan Africa: a mixed methods study. Int J Ment Health Syst.

[76] McInnes E, Wimpenny P (2008). Using qualitative assessment and review instrument software to synthesise studies on older people's views and experiences of falls prevention. Int J Evid Based Healthc.

[77] McIntosh K, Mercer SH, Nese RN, Strickland-Cohen MK, Kittelman A, Hoselton R, Horner RH (2018). Factors predicting sustained implementation of a universal behavior support framework. Educ Res.

[78] Mazzucchelli TG, Sanders MR (2010). Facilitating practitioner flexibility within an empirically supported intervention: lessons from a system of parenting support. Clin Psychol.

[79] Stirman SW, Kimberly J, Cook N, Calloway A, Castro F, Charns M (2012). The sustainability of new programs and innovations: a review of the empirical literature and recommendations for future research. Implement Sci.

[80] Bergdahl E (2019). Is meta-synthesis turning rich descriptions into thin reductions? A criticism of meta-aggregation as a form of qualitative synthesis. Nurs Inq.

[81] Bearman M, Dawson P (2013). Qualitative synthesis and systematic review in health professions education. Med Educ.

