# Supplementary material for: Delivering and implementing child and adolescent mental health training for mental health and allied professionals: a systematic review and qualitative meta-aggregation
Source: BMC Med Educ. 2021 Feb 15;21:103. doi: 10.1186/s12909-021-02530-0 (PMC7885386; doi:10.1186/s12909-021-02530-0)
Supplement: Supplementary file 1 — Additional file 1. [file 12909_2021_2530_MOESM1_ESM.pdf]

The list of search terms is divided into the following categories. Three example terms have been provided below for each:

1) Population:
   a) *Roles of trainees*, e.g. nurses; psychologists; police
   b) *Conditions/disorders focussed on,* e.g. depression; anxiety; psychosis
   c) *Condition/disorder associated terms,* e.g. disorder; difficulty; condition
   d) *Population training focusses on,* e.g. children; teenagers; pupils
2) Focal areas:
   a) *Training,* e.g. course; professional development; awareness
   b) *Focus of literature,* e.g. opinions; effectiveness; evaluation
3) Data generation methods, e.g. qualitative; interview; audit

Terms will be truncated where necessary, e.g. child* to cover child; child's; children; children's'; childhood.

Example search strategy using Scopus (ordered by specificity of fields searched):

Focus of training:

TITLE(child* OR teen* OR adolescen* OR youth* OR "young people" OR "young person*" OR "young adult*" OR young OR CYP OR minor* OR juvenile* OR school OR college OR pupil* OR student* OR "you* offend*" OR paediatric* OR "looked after*" OR toddler* OR bab* OR boy* OR girl* OR LGBT*)

Training:

AND TITLE(training OR traine* OR "learning package*" OR course* OR "learning resource*" OR skills OR "skills development" OR "professional development" OR "staff development" OR knowledge OR education OR awareness OR "policy change")

Roles of trainees:

AND TITLE-ABS("mental health professional*" OR "allied professional*" OR "professional*" OR staff OR employee* OR nurse* OR paediatric* OR doctor* OR practitioner* OR GP* OR psycholog* OR psychiatr*OR teacher* OR counsell* OR "social worker*" OR "youth worker*" OR "support worker*" OR police OR paramedic* OR "prison officer*")

Conditions/disorders focussed on:

AND TITLE-ABS(depress* OR anxi* OR schizophreni* OR bipolar OR psych* OR mental* OR OCD OR obsessive-compulsive OR "obsessive compulsive" OR self-harm* OR emotion* OR "self harm*" OR suicid* OR "eating disorder*"OR "disordered eating" OR anorexi* OR bulimi*OR autis* OR ADHD OR "attention deficit hyperactivity" or hyperactive* OR hyperkinetic OR conduct OR panic)

Condition/disorder associated terms:

AND TITLE-ABS("mental health" OR wellbeing OR disorder* OR illness* OR crisis OR difficult* OR condition* OR problem*)

Focus of literature:

AND TITLE-ABS(barrier* OR facilitat* OR implement* OR sustain* OR intervention* OR effective* OR success* OR evaluat* OR apprais* OR assess* OR review* OR reflect* OR view* OR observ* OR perspective* OR perception* OR opinion* OR experience* OR story OR stories OR narrative* OR theme*)

Data generation methods:

AND ALL(qualitativ* OR “mixed method*” OR interview* OR “focus group*” OR observation* OR ethnograph* OR “case stud*” OR “action research” OR “document review” OR audit)
